# *Staphylococcus aureus* utilizes environmental RNA as a building material in specific polysaccharide-dependent biofilms

**DOI:** 10.1038/s41522-022-00278-z

**Published:** 2022-04-04

**Authors:** Akio Chiba, Masahide Seki, Yutaka Suzuki, Yuki Kinjo, Yoshimitsu Mizunoe, Shinya Sugimoto

**Affiliations:** 1 Department of Bacteriology, The Jikei University School of Medicine, 3-25-8 Nishi-Shimbashi, Minato-ku, Tokyo, 105-8461 Japan; 2 Jikei Center for Biofilm Science and Technology, The Jikei University School of Medicine, 3-25-8 Nishi-Shimbashi, Minato-ku, Tokyo, 105-8461 Japan; 3 Department of Computational Biology and Medical Sciences, Graduate School of Frontier Sciences, The University of Tokyo, 5-1-5 Kashiwanoha, Kashiwa, Chiba, 277-8561 Japan

**Keywords:** Clinical microbiology, Sequencing, Biofilms, Microbial communities

## Abstract

Biofilms are surface-bound microbial communities that are typically embedded in a matrix of self-produced extracellular polymeric substances and can cause chronic infections. Extracellular DNA is known to play a crucial role in biofilm development in diverse bacteria; however, the existence and function of RNA are poorly understood. Here, we show that RNA contributes to the structural integrity of biofilms formed by the human pathogen *Staphylococcus aureus*. RNase A dispersed both fresh and mature biofilms, indicating the importance of RNA at various stages. RNA-sequencing analysis demonstrated that the primary source of RNA in the biofilm matrix was the Brain Heart Infusion medium (>99.32%). RNA purified from the medium promoted biofilm formation. Microscopic and molecular interaction analyses demonstrated that polysaccharides were critical for capturing and stabilizing external RNA in biofilms, which contributes to biofilm organization. These findings provide a basis for exploring the role of externally derived substances in bacterial biofilm organization.

Biofilms are highly organized communities of microbes that attach to surfaces to survive in diverse environments^1,2^. Nearly microbes of every species have mechanisms by which they can adhere to biotic or abiotic surfaces and to each other, allowing them to exist in biofilms^3^. Microbial cells within a biofilm are embedded in a matrix of self-produced extracellular polymeric substances, including proteins^4–6^, polysaccharides^7–10^, and/or DNA^11,12^, which allow them to evade the host immune system and antimicrobial agents^13–15^. In addition, biofilms promote the transfer of genetic information, thereby conferring antibiotic resistance^16^. Thus, biofilms formed on the surfaces of medical implants and host organism tissues can cause chronic biofilm-associated infections, including catheter-related bloodstream infections, urinary tract infections, and endocarditis^17,18^. Knowledge of how these communities develop is important for their eradication; however, the mechanistic basis for biofilm formation remains incompletely understood at the molecular level. Identifying biofilm matrix components that contribute to biofilm structural integrity can provide insight into the process of biofilm development that can lead to the development of strategies for the inhibition of biofilm formation.

*Staphylococcus aureus* is a major biofilm-forming Gram-positive bacterium^19^. Approximately one-third of healthy people are asymptomatically colonized by *S. aureus*, a microbe known to cause infections^20^. Owing to its ability to form biofilms on medical devices, including indwelling catheters, artificial heart valves, and endotracheal tubes, *S. aureus* often causes chronic or fatal infections in clinical settings. Matrix components of *S. aureus* biofilms typically include proteins^21,22^, polysaccharides^9^, and/or DNA^23^ produced by the bacterial cells themselves. Despite extensive research on these components in various bacterial biofilms^24^, little is known about RNA-dependent biofilms. A report has revealed that a ribonuclease can inhibit biofilm formation by *Haemophilus influenzae*^25^, implying that RNA is present in a biofilm, although direct evidence is lacking. In addition, the origin of this RNA, as well as its incorporation into biofilms and stable existence in them, remain unknown.

In this study, we found that most of the RNAs originated from the culture medium, and the external RNA contributed to robust biofilm organization via interaction with polysaccharides. These findings provide evidence of a function for RNA that can lead to the development of strategies to prevent biofilm formation.

## Results

### RNA is important for maintaining biofilm structural scaffolds

**Fig. 1:**
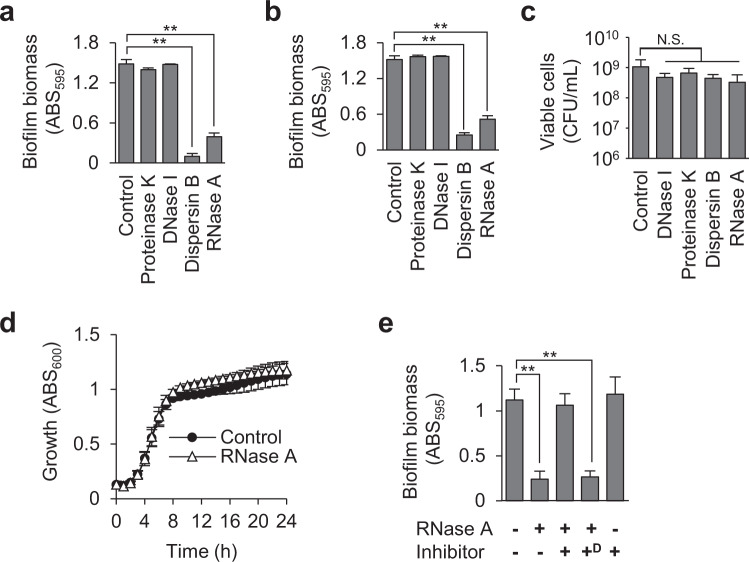
Extracellular RNA maintains biofilm structural scaffolds. **a** *Staphylococcus aureus* MR10 24-h biofilms were treated with the indicated enzymes for 30 min at 37 °C or left untreated (Control). **b** MR10 biofilms were formed in the absence (Control) or presence of the indicated enzymes for 24 h at 37 °C. **c** The numbers of viable cells in the 24-h biofilms formed in the absence (Control) or presence of the indicated enzymes were counted as colony-forming units (CFUs) per 1 mL. **d** The growth of MR10 was measured in BHIN with or without RNase A (80 μg/mL) in a 96-well plate under static conditions. The absorbance of each well at 600 nm was measured with an incubation reader (Scinics Corporation, Tokyo, Japan). **e** MR10 24-h biofilms were treated or not with RNase A. RNase inhibitor was added (+). In addition, the RNase inhibitor was denatured at 105 °C for 5 min and then added (+^D^). Biofilm biomasses are presented as the mean and standard deviation (error bar) of three independent experiments. **P < 0.01. N.S. not significance.

To identify the major components of biofilm matrices, we evaluated the susceptibility of *S. aureus* biofilms to various enzymes. MR10, a clinically isolated methicillin-resistant *S. aureus* strain that forms a robust polysaccharide-enriched biofilm^26^ (Table 1), was cultured in Brain Heart Infusion (BHI) medium supplemented with 3% NaCl (BHIN) to form biofilms. The biofilm was destroyed by dispersin B, a β-1-6 N-acetylglucosaminidase that cleaves extracellular polysaccharides produced by staphylococci^27^, and by RNase A (Fig. 1a). In contrast, neither DNase I nor proteinase K destroyed the mature biofilm (Fig. 1a). Similarly, RNase A and dispersin B, but neither DNase I nor proteinase K, inhibited biofilm formation by the strain without growth inhibition and loss of viability under the tested conditions (Fig. 1b–d). In addition, RNase A destroyed the biofilm of RN4220^28^, a laboratory strain of methicillin-sensitive *S. aureus* that formed a polysaccharide-dependent biofilm, and inhibited its formation (Supplementary Fig. 1a, b), indicating that the observed phenomena were not limited to strain MR10. In contrast, a polysaccharide-independent biofilm of strain USA300^29^ was neither inhibited nor dispersed but promoted by RNase A via unknown mechanisms (Supplementary Fig. 1c, d).

**Table 1:** Bacterial strains and plasmids used in this study.

Name	Description^a^	Source and reference
*Staphylococcus aureus* strains		
MR10	A clinical isolate of MRSA from the Jikei hospital	26
MR10 Δ*icaA*	The Δ*icaA* isogenic strain of MR10	This study
RN4220	A restriction deficient derivative of 8325-4 used for sub-cloning host	28
USA300	A clinical isolate of MRSA	29
MR3	A clinical isolate of MRSA from the Jikei hospital	31
MS7	A clinical isolate of MSSA from the Jikei hospital	31
MS8	A clinical isolate of MSSA from the Jikei hospital	31
MS9	A clinical isolate of MSSA from the Jikei hospital	31
MS12	A clinical isolate of MSSA from the Jikei hospital	31
SH1000	8325-4 with functional *rsbU*	30
*Escherichia coli* strain		
DH5α	*fhuA2* Δ*(argF-lacZ)U169 phoA glnV44 Φ80* Δ*(lacZ)M15 gyrA96 recA1 relA1 endA1 thi-1 hsdR17*	BioDynamics Laboratoy (Tokyo, Japan)
Plasmids		
pGEM-T Easy Vector	Amp^R^, M13*ori* pBR322*ori*, linear-T-overhand vector	Promega
pKOR1	An *E. coli*-*S. aureus* shuttle vector plasmid for knockout of staphylococcal genes by allelic exchange, Cm^R^, Amp^R^	43
pKOR1-icaAKO	A pKOR1-derivative plasmid for knockout of the *icaA* gene in MR10, Cm^R^, Amp^R^	This study
pLC1	An *E. coli*-*S. aureus* shuttle vector plasmid, Cm^R^, Amp^R^	48
pLC1::*ica*	pLC1-derivative plasmid for expression of *ica* operon containing the ribosome-binding site of *icaA*, Cm^R^, Amp^R^	This study

^a^Amp^R^, ampicillin resistance; Cm^R^, Chloramphenicol resistance.

Next, we tested whether RNase A disperses various polysaccharide-dependent biofilms of *S. aureus*. For this purpose, we used a laboratory strain SH1000^30^ and 5 clinically isolated strains that formed polysaccharide-dependent biofilms in BHIN^31^. As shown in Supplementary Fig. 1e, several but not all strains showed sensitivity against RNase A, indicating that RNA plays an important role in specific polysaccharide-dependent biofilms.

To determine whether biofilm destruction by RNase A was due to its ribonucleolytic activity, we abolished the latter by applying the proteinaceous RNase inhibitor RNasin^32^. RNase A did not destroy preformed biofilms in the presence of RNasin but did so when heat-denatured RNasin was added (Fig. 1e), indicating that the ribonucleolytic activity of RNase A is required for biofilm destruction. These results suggest that RNA is present in biofilms and contributes to their structural integrity.

**Fig. 2:**
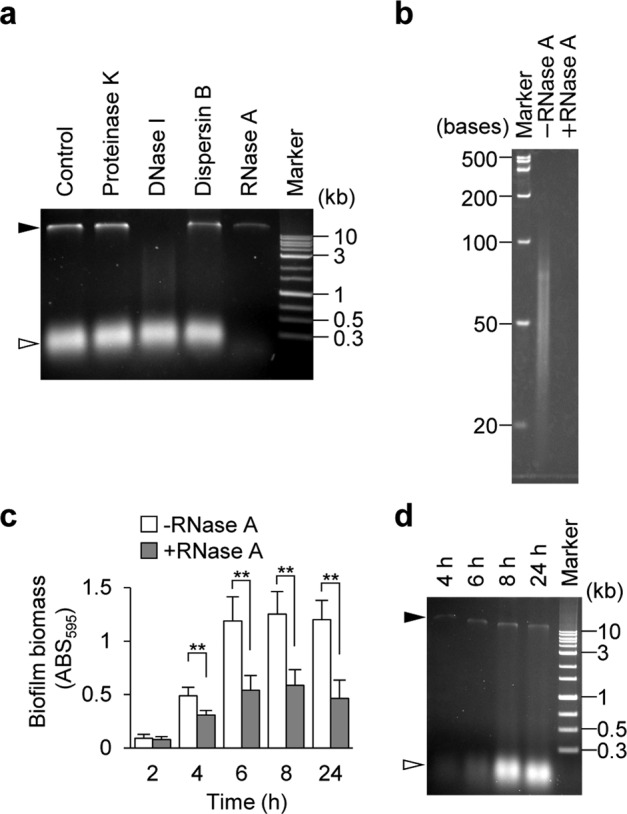
Extracellular RNA is present in the *Staphylococcus aureus* biofilm matrix. **a** A biofilm matrix extracted from 24-h biofilms of MR10 was treated with the indicated enzymes or left untreated (Control); nucleic acids were separated on agarose gels. **b** RNA size was estimated using urea-denaturing polyacrylamide gel electrophoresis. The sample after treatment with RNase A was also loaded as a control. **c** MR10 biofilms formed at the indicated time points (hours) were treated with RNase A (gray bars) or left untreated (white bars) with RNase A. The data are presented as the mean and standard deviation (error bar) of three independent experiments. **P < 0.01. **d** A biofilm matrix was isolated from the biofilms at the indicated time points (hours) and the presence of nucleic acids was determined using agarose gel electrophoresis. The applied volume was standardized by biofilm wet weight (10 μL of the biofilm matrix extracted from 0.44 mg of the biofilm was applied per one lane). The white and black arrowheads denote RNA and DNA, respectively.

To identify the presence of RNA in a biofilm matrix, we isolated the biofilm matrix from MR10 using our established technique^26^, which enabled us to peel off the matrix from the surface of bacterial cells using a high ionic-strength solution containing 1.5 M NaCl and analyzed the nucleic acid content by using agarose gel electrophoresis. Two bands of >10 kb and approximately 300 bp were detected (Fig. 2a). The high molecular weight band was degraded by DNase I (Fig. 2a), indicating that it was composed of DNA. In contrast, the low-molecular-weight band disappeared following treatment with RNase A, but not DNase I (Fig. 2a), indicating that it was RNA. Denaturing polyacrylamide gel electrophoresis was performed to accurately determine band size; smears ranging from 20 to 100 bases disappeared upon RNase A treatment (Fig. 2b).

To determine whether RNA is involved in a particular stage of biofilm development, we examined the susceptibility of MR10 biofilms to RNase A treatment over time. RNase A destroyed both fresh and mature biofilms over a period of 4–24 h (Fig. 2c). We analyzed the matrix extracted from biofilms at the indicated culture times using agarose gel electrophoresis. RNA was detected in the 4-h biofilm, and the intensity of the band increased throughout biofilm formation (Fig. 2d). These results suggest that RNA is utilized at various stages of biofilm formation under the tested conditions.

### External RNA is utilized for biofilm formation

**Fig. 3:**
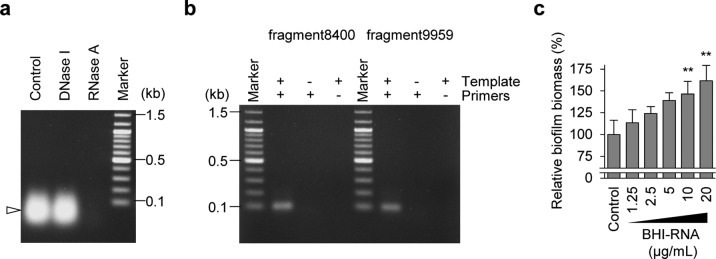
RNA from the surrounding milieu affects biofilm formation. **a** Nucleic acids were isolated from BHI medium via ethanol precipitation, treated with DNase I and RNase A or left untreated (Control), and detected on an agarose gel. A white arrowhead denotes RNA. **b** The presence of RNA identified by RNA-seq was confirmed by reverse transcriptase-polymerase chain reaction using RNA purified from the BHI medium as a template and primers specific for the fragments identified through RNA-seq (Supplementary Table 1). + added, − not added. **c** The effects of the RNA purified from BHI medium (BHI–RNA) on the formation of MR10 biofilms in Roswell Park Memorial Institute medium supplemented with 1% glucose. The data are presented as the mean and standard deviation (error bar) of relative biofilm biomass from triplicate experiments. The biofilm biomass in the absence of BHI–RNA was defined as 100%. **P < 0.01.

We performed RNA-seq analysis to investigate the source of RNA in the biofilm matrix of MR10. Mapping the reads to the publicly available *S. aureus* Mu50 genome sequence^33^ revealed that RNA derived from *S. aureus* represented only 0.68% (110,111 counts) of the total (16,239,718 counts), and almost all of them were assigned to tRNAs and rRNAs (Supplementary Fig. 2a). To investigate the possibility that external RNA was incorporated into the biofilm matrix, we examined the RNA present in the culture environment, including nucleic acids, in the BHI medium. The ethanol-precipitated BHI medium contained RNase A-sensitive but DNase I-resistant RNA (Fig. 3a) that migrated on an agarose gel in a pattern similar to that observed for biofilm matrices (Fig. 2a), indicating that BHI medium was the major source of RNA in the biofilm matrix. We then assessed whether the RNA detected by RNA-seq was identical to that present in BHI medium using reverse transcriptase (RT)-PCR amplification of two non-*S. aureus* sequences identified by RNA-seq (Table 2). Two sets of specific primers (Supplementary Table 1) and RNA purified from the BHI medium were added to the reaction, and 100-base fragments of the expected sizes were amplified (Fig. 3b). Sequence analysis revealed that the amplified fragments had the same sequence as those identified by RNA-seq. We used a similar procedure to confirm the presence of RNA derived from *S. aureus*. cDNA corresponding to the RNA fragment, identified via RNA-seq (Supplementary Fig. 2b), was amplified using RNA purified from the biofilm-matrix fraction of MR10 as a template (Supplementary Fig. 2c). In contrast, no product was obtained when the template consisted of RNA purified from the BHI medium (Supplementary Fig. 2c). These results demonstrate that *S. aureus* RNA is present in the biofilm matrix, but the majority of RNA in the biofilm is derived from the environment. In addition, we performed RNA-seq analysis to compare the sequences of RNAs purified from BHI medium with those from the biofilm-matrix fraction of MR10. As shown in Supplementary Fig. 3, many abundant RNA sequences in the medium were also detected in the biofilm-matrix fraction. The composition of the RNA in the biofilm matrix reflected the concentration of the RNA in the medium.

**Table 2:** The selected sequence was used as the template for RT-PCR.

Sequence name	Sequence (from 5′ to 3′)^a^
fragment9959_count111_len101	GAAAGCACCG TTTCCCGTCC GATCAACTGT AGTTAAGCTG GTAAGAGCCT GACCGAGTAG TGTAGTGGGT GACCATACGC GAAACTCAGG TGCTGCAATC T
fragment8400_count133_len100	TCATTTGTAT ACGACTTAGA TGTACAACGG GGTATTGTAA GCAGTAGAGT AGCCTTGTTG TTACGATCTG CTGAGATTAA GCCTTTGTTG TCTGATTTGT

^a^Among the reads obtained by RNA-seq, long sequences (>100 nt) with more than 100 counts were selected to confirm whether these RNAs were derived from BHI medium. The fragments were amplified by RT-PCR, and the products were analyzed using agarose gel electrophoresis (Fig. 3b).

**Fig. 4:**
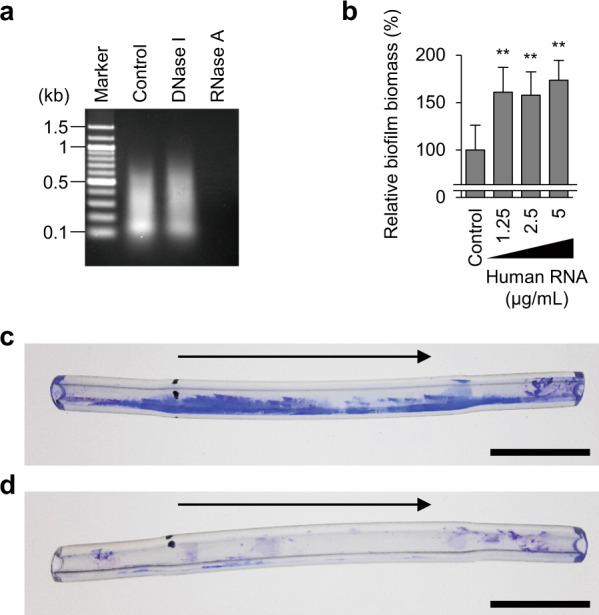
Effects of human RNA on biofilm formation. **a** A profile of human RNA isolated from total blood using ISOGEN-LS is shown. The purified RNA was treated with the indicated enzymes or left untreated (Control) and subsequently analyzed using agarose gel electrophoresis. **b** The effects of human RNA on biofilm formation by MR10 in RPMIG. Biofilm biomass is shown as mean and standard deviation (error bar) from three independent experiments. **P < 0.01. **c, d** Biofilm formation in polyurethane tubes was analyzed under continuous flow conditions. MR10 was grown in PNG supplemented with (**c**) or without human RNA (**d**). Representative images of three independent experiments are shown. The arrows indicate the directions of flow. Scales indicate 1 cm.

We hypothesized that external RNA could be used as a building block in biofilms. We confirmed the biofilm-promoting effect of RNA purified from BHI medium (BHI–RNA) using Roswell Park Memorial Institute medium supplemented with 1% glucose (RPMIG), which does not contain any nucleic acids. BHI–RNA promoted biofilm formation by MR10 cells in RPMIG (Fig. 3c). Next, human RNA was purified from human total blood (Fig. 4a) and tested to promote biofilm formation by MR10 in RPMIG medium. As shown in Fig. 4b, human RNA significantly promoted biofilm formation. In addition, we investigated the promoting effect of human RNA under flow conditions using plastic tubes made from polyurethane, a material generally used for intravascular catheters. In this assay, we used the peptone–NaCl–glucose (PNG) medium that did not contain RNA and has frequently been used in the study of *S. aureus* biofilms^34^. When human RNA was added to the medium, biofilm formation in the tube was promoted (Fig. 4c, d). Collectively, these data suggest that external RNA could be used for biofilm development by this human pathogen during infection and colonization.

### Extracellular polysaccharides maintain and stabilize RNA

**Fig. 5:**
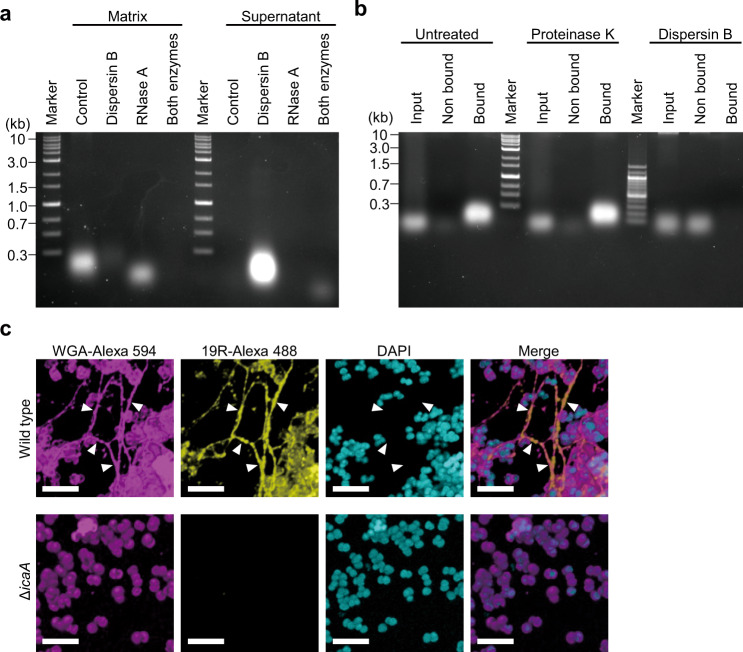
Polysaccharides capture external RNA in the biofilm. **a** A biofilm matrix was extracted from MR10 24-h biofilms formed in BHIN. Dispersin B alone, RNase A alone, or both enzymes were added to the biofilm immediately before the biofilm-matrix extraction. After incubation for 1 h at 37 °C, the treated samples were centrifuged. After centrifugation, the supernatants were collected and the biofilm matrices were extracted from the pellets using 1.5 M NaCl. The nucleic acids were analyzed using agarose gel electrophoresis. **b** RNA pull-down was performed as illustrated in Supplementary Fig. 4. Biofilm-associated and nonassociated RNA from MR10 wild type pretreated with proteinase K and dispersin B or left untreated were detected using agarose gel electrophoresis. **c** The biofilms of MR10 wild-type and its isogenic Δ*icaA* strains were cultured in BHINC supplemented with 19R-Alexa 488 for 24 h at 37 °C. The biofilms were stained with WGA-Alexa 594 and DAPI. Subsequently, the biofilms were observed by high-resolution CLSM (with Airyscan unit). WGA-Alexa 594 and DAPI stained the polysaccharides and the cell wall (magenta) and DNA (cyan), respectively. 19R-Alexa 488 indicates localization of RNA (yellow). The merged images are also shown. Scale bars correspond to 5 µm. The arrowheads indicate colocalization of polysaccharides and RNA.

RNA is generally assumed to be unstable and readily degraded. However, the presence of RNA in bacterial biofilm matrices suggests that it is stable in this environment. Our results showed that various polysaccharide-dependent biofilms were dispersed by RNase A (Fig. 1 and Supplementary Fig. 1). Based on these results, we speculated that structural scaffolds, such as polysaccharides within biofilms, bind to and stabilize RNA. To test this hypothesis, MR10 biofilms formed in BHIN were treated with dispersin B immediately before biofilm-matrix extraction and then separated using agarose gel electrophoresis. The band corresponding to RNA (Fig. 2a) was detected in the supernatant but not in the matrix of the dispersin B-treated biofilm (Fig. 5a). When the biofilm was treated with RNase A, instead of dispersin B, the band intensity of RNA in the matrix did not disappear completely (Fig. 5a). Moreover, when the biofilm was treated with both RNase A and dispersin B, only a faint band was detected in the supernatant, and no band was observed in the matrix (Fig. 5a). These results suggest that polysaccharides protect the RNA in the biofilm.

In addition, we examined whether extracellular polysaccharides play a role in capturing environmental RNA in biofilms. Biofilms were mixed with an RNA solution prepared from the filtrate of cultured BHI medium, and the supernatant was analyzed using agarose gel electrophoresis to detect nonbound RNA. The input and biofilm-associated fractions (containing bound RNA) were also examined (Supplementary Fig. 4). RNA molecules were captured by untreated wild-type and proteinase K-treated MR10 cells, but not by dispersin B-treated cells or by the Δ*icaA* strain (Fig. 5b and Supplementary Fig. 5a) that is unable to produce polysaccharides^9,10^. Transcomplementation of Δ*icaA* with the *icaADBC*-expression plasmid recovered biofilm biomass (Supplementary Fig. 5b), enzyme susceptibility (Supplementary Fig. 5c, d), and RNA binding (Supplementary Fig. 5a). These results imply that polysaccharides, but not cell-surface proteins, directly or indirectly capture RNA in biofilms.

To observe the localization of RNA in the biofilm of MR10, we synthesized a medium-derived abundant RNA (termed 19 R, CGUUAGUUCAAUUCUGACA). RNA-seq data revealed that 19 R was included in 16.47% of the total reads. In addition, we confirmed that 19 R promoted biofilm formation in RPMIG in a concentration-dependent manner (Supplementary Fig. 6). Next, we observed the colocalization of RNA and polysaccharides in biofilms by confocal laser-scanning microscopy (CLSM) using synthesized Alexa Fluor 488-labeled 19 R (19R-Alexa 488) and Alexa Fluor 594-conjugated lectin wheat germ agglutinin (WGA-Alexa 594). Given that WGA recognizes N-acetylglucosamine, it can bind to polysaccharides and the cell wall^35^. In this experiment, BHIN was treated with 10% activated charcoal (BHINC) to remove free RNA from BHIN (Supplementary Fig. 7a), which enabled us to prevent free RNA in BHIN from interfering with the interaction between fluorescence-labeled RNA and polysaccharides. Importantly, the major component of the biofilm formed in BHINC was polysaccharides, as the biofilm was destroyed by dispersin B but not by the other enzymes (Supplementary Fig. 7b). Biofilm biomass was lower in BHINC than in BHIN (Supplementary Fig. 7b), and supplementation of 19 R into BHINC promoted biofilm formation in a concentration-dependent manner (Supplementary Fig. 7c), underscoring the role of environmental RNA as a key determinant of biofilm organization. RNA colocalized with extracellular polysaccharide networks in wild-type but not in Δ*icaA* biofilms (Fig. 5c and Supplementary Fig. 8a). Image analysis for colocalization of RNA and polysaccharides revealed that 19–82% of polysaccharides colocalized with RNA (Supplementary Fig. 9). Transcomplementation of Δ*icaA* with the *icaADBC*-expression plasmid recovered RNA colocalization (Supplementary Fig. 8b). These results suggest that external RNA associates directly or indirectly with biofilm polysaccharides.

**Fig. 6:**
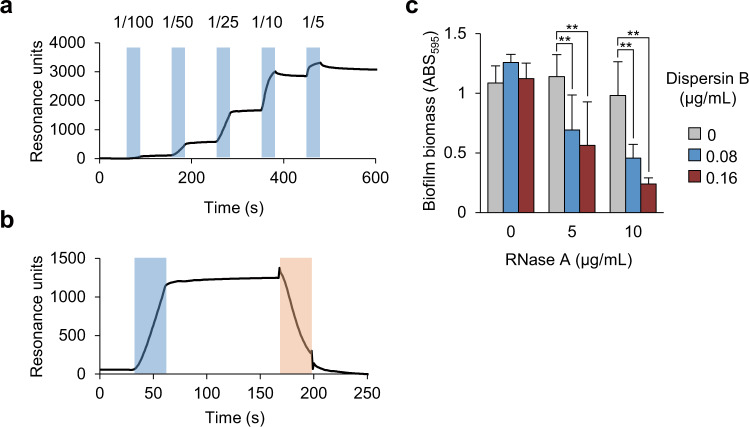
Direct interaction between RNA and polysaccharides. **a**, **b** Interaction analysis between RNA and purified polysaccharides was performed using surface plasmon resonance. The biotinylated 19 R was immobilized on a streptavidin-coated sensor chip. **a** Purified polysaccharides were diluted to the indicated folds and were applied to the flow cell during the time points (second) denoted in light blue. **b** Purified polysaccharides were diluted to 25-fold and were applied to the flow cell during the times denoted in light blue. Subsequently, diluted dispersin B (16 µg/mL) was applied during the times denoted in orange. **c** The 24-h MR10 biofilms were treated with the indicated concentrations of RNase A and/or dispersin B for 60 min at 37 °C. To avoid the destruction of the preformed biofilms, low concentrations of RNase A and dispersin B alone were used. The data are presented as the mean and standard deviation (error bar) of three independent experiments. **P < 0.01.

We investigated whether there is a direct interaction between RNA and polysaccharides, using surface plasmon resonance analysis, 19 R, and polysaccharides purified from MR10 biofilms. 19 R was immobilized on a sensor chip as a ligand. The purified polysaccharides were applied to the flow cell as the analytes. We observed that the polysaccharides bound RNA in a dose-dependent manner (Fig. 6a). In addition, dispersin B treatment caused a decrease in the sensorgram due to the degradation of polysaccharides bound to the immobilized RNA (Fig. 6b). These results confirmed the direct binding of RNA to polysaccharides.

To clarify the biological significance of the interaction between RNA and polysaccharides, we investigated the effects of combined RNase A and dispersin B treatment on biofilm formation. When administered alone at low concentrations, neither of the enzymes dispersed mature biofilms (Fig. 6c). However, this was achieved by incubation with both enzymes, even at low concentrations (Fig. 6c). These results suggest that polysaccharides and RNA engage in mutual stabilization, leading to robust biofilm organization. This was consistent with our observation that polysaccharides protected RNA from degradation in biofilms (Fig. 5a).

## Discussion

RNA in the form of mRNA, tRNA, and noncoding regulatory RNAs is critical for the transfer of genetic information in cells^36,37^. In bacteria, noncoding RNAs regulate transcription and translation and affect biofilm phenotype^38^. Here, we report a function of RNA: RNA acts in cooperation with polysaccharides to promote bacterial biofilm formation.

A ribonuclease was reported to inhibit the formation of *H. influenzae* biofilm but did not disperse the preformed biofilm^25^, however, RNA was not detected in the *H. influenzae* biofilm. In addition, these authors did not describe the characteristics and source of the ribonuclease used in the study. Certain ribonucleases are known to inhibit bacterial growth^39^; therefore, it is unclear whether the observed biofilm-inhibitory effect was due to bacterial growth suppression or degradation of biofilm scaffolds composed of RNA. Here, we identified and characterized RNA in *S. aureus* biofilms and demonstrated that RNase A can inhibit biofilm formation by *S. aureus* without causing growth inhibition and loss of cell viability (Fig. 1b–d) and destroy preformed *S. aureus* biofilms via its ribonucleolytic activity (Fig. 1e). Therefore, to the best of our knowledge, this is the first report showing that RNA exists in the biofilm matrices and contributes to the robustness of *S. aureus* biofilms.

Thus far, the underlying mechanism of RNA incorporation into *S. aureus* biofilms has been unknown; probably, there is involvement of multiple mechanisms. We propose that at least one mechanism driven by the interaction between RNA and polysaccharides is important (Figs. 5 and 6). A recent study suggested that extracellular polysaccharides might interact with eDNA in an *S. aureus* biofilm^40^; however, a direct interaction between polysaccharides and DNA has not been demonstrated so far. In the current study, using surface plasmon resonance analysis, we showed that purified extracellular polysaccharides directly interacted with RNA (Fig. 6a). This is the first report of bacterially produced exopolysaccharides that directly bind to RNA to promote biofilm organization. This interaction may protect RNA from degradation in the environment. The synergistic effects of dispersin B and RNase A on the degradation of RNA and dispersal of a preformed biofilm were observed (Figs. 5a and 6c), suggesting that extracellular polysaccharides could stabilize RNA in the biofilm. In addition, our data showed that certain RNAs in BHI medium were enriched in the biofilm-matrix fraction of MR10 (Supplementary Fig. 3). Polysaccharides may selectively bind to specific RNAs in the medium. Further studies are required to elucidate the molecular mechanisms underlying the enrichment of certain RNAs in the biofilm matrix.

Given that diverse *S. aureus* clinical strains formed DNA-dependent biofilms and the formation of polysaccharide-specific biofilms was found in certain clinical strains^31^, RNA-dependent biofilms could be more strain-specific than DNA-dependent ones. Our data indicated that RNA plays a key role in specific polysaccharide-dependent biofilms, while a few strains formed polysaccharide-dependent but RNase A-resistant biofilms. Our previous data indicated that MR10 produced a large amount of polysaccharides and the levels of polysaccharides differed among the strains^31^. Differences in the levels of PIA and extracellular nucleases that degrade environmental RNA may reflect the RNA dependency.

RNA purified from human blood promoted biofilm formation (Fig. 4b). Compared with BHI–RNA and 19 R RNA, the addition of human RNA only resulted in a modest increase in biofilm biomass. We speculate that there are some sequence specificities of RNA for promotion of biofilm formation. In addition, length of RNA may also affect this function, since various longer fragments were detected in human RNA, while only short fragments were detected in RNA purified from BHI. These differences may account for the distinct effects on biofilm formation between human RNA and the others.

Interestingly, human blood RNA at physiological concentrations^41^ promoted biofilm formation under flow conditions (Fig. 4c). The result suggests that RNA may serve as a biofilm scaffold during infection. *S. aureus* is one of the major causative bacteria in intravascular infections, including catheter-related bloodstream infections, and infective endocarditis. *S. aureus* invading the vessel might utilize blood RNA to form a strong biofilm on implanted medical devices or tissues, causing chronic infections. In addition, other bacteria could adopt the same strategy during infection.

Taken together, these findings provide new insights into bacterial adaptation strategies, whereby bacterially produced extracellular polymeric substances cooperate with available materials in the extracellular milieu in the construction of a bacterial habitat. Most previous studies have primarily focused on bacterially produced biofilm-matrix components; however, our findings indicate that environmental substances are incorporated into the matrix, possibly in a species- and/or strain-specific manner, and contribute to biofilm organization. Our report will open the door to extracellular functions of RNA beyond the transfer of genetic information and future studies will address the role of environmental RNA in biofilms of other bacteria.

## Methods

### Bacterial strains and culture media

The bacterial strains used in this study are listed in Table 1. Brain Heart Infusion (BHI) medium (Becton Dickinson, Franklin Lakes, NJ), BHI medium supplemented with 1% (w/v) glucose (Wako, Osaka, Japan) (BHIG), BHI medium supplemented with 3% (w/v) NaCl (Wako) (BHIN), tryptic soy broth (TSB) (Becton Dickinson), or peptone–NaCl–glucose (PNG) [0.33% (w/v) peptone (Becton Dickinson), 0.26% (w/v) sodium chloride, and 0.33% glucose (w/v)] were autoclaved at 121 °C for 15 min. If required, BHIN was mixed with 10% (w/v) activated charcoal and filtered using a 0.2-µm pore filter (Kanto Chemical, Tokyo, Japan), and the filtrate BHINC was used to culture the biofilms. RPMI medium (Thermo Fisher Scientific, Carlsbad, CA) was supplemented with 1% glucose (w/v) (RPMIG) and passed through a 0.2-µm pore filter. Lysogeny-broth (LB) medium (Millipore, Bedford, MA) was used to culture *Escherichia coli*. All the bacterial strains were grown in the appropriate media at 37 °C.

### Construction of gene-deletion strain

Approximately 500 bp upstream and downstream sequences of *icaA* from MR10 genomic DNA were amplified using the polymerase chain reaction (PCR) with the following primer sets: icaA_F, SOE-icaA-R, and icaA_R; SOE-icaA-F (see Supplementary Table 1). These fragments were ligated by splicing using an overlap extension PCR^42^. The resulting fragment was cloned into pKOR1 using Gateway BP Clonase II enzyme mix (Thermo Fisher Scientific), and the resulting plasmid was named pKOR1-icaAKO (Table 1). Using pKOR1-icaAKO, *icaA* was deleted from the MR10 genomic DNA by in-frame deletion according to a previously reported procedure^43^. Briefly, pKOR1-icaAKO was used to transform *S. aureus* RN4220^28^ through electroporation. After purification from RN4220, the plasmid was introduced into MR10 through electroporation. The transformant harboring pKOR1-icaAKO was cultured on NYE agar (1% casein hydrolysate, 1% yeast extract, and 0.5% NaCl, pH 7.2) containing 5 μg/mL chloramphenicol (Cm) (Nacalai) at 30 °C for 24 h. A single colony on the plate was inoculated into BHI containing 5 μg/mL Cm and grown at 30 °C for 48 h. The culture was diluted 1000-fold into BHI containing 5 μg/mL Cm and incubated at 42 °C overnight. The overnight culture (50 μL) was spread on TSB agar containing 20 μg/mL Cm and incubated at 42 °C overnight. A single colony was inoculated into TSB and grown at 30 °C overnight. The overnight culture was diluted 100-fold into TSB, and the aliquot (100 μL) was spread on TSB agar containing 100 ng/mL anhydrotetracycline. After incubation at 30 °C overnight, several big colonies on the plate were selected as the MR10 Δ*icaA* candidates and grown in TSB at 37 °C overnight. Deletion of *icaA* was confirmed by genomic PCR.

### Reagents

Phosphate-buffered saline (PBS) was purchased from Nissui Pharmaceutical (Tokyo, Japan), and crystal violet (CV) and ISOGEN-LS were purchased from Wako; DAPI was purchased from Dojindo (Kumamoto, Japan); and wheat germ agglutinin–Alexa Fluor 594 (WGA-Alexa594) was purchased from Thermo Fisher Scientific. Oligoribonucleotides were synthesized by Gene Design (Osaka, Japan), and oligodeoxyribonucleotides were synthesized by Thermo Fisher Scientific.

### Degradative enzymes

Dispersin B (Kane Biotech Inc., Winnipeg, Canada), proteinase K (Sigma, St. Louis, MO), DNase I (protease- and RNase-free, Roche Diagnostics, Mannheim, Germany), and RNase A (protease- and DNase-free, Nacalai Tesque, Kyoto, Japan) were used in this study. The appropriate buffer [40 mM Tris-HCl (pH 7.9), 10 mM NaCl, 6 mM MgCl_2_, and 1 mM CaCl_2_] was used for DNase-I treatment according to the manufacturer’s instructions, while the other enzymes were added to biofilm cultures without buffer. The final enzyme concentrations and reaction times are described below.

### Bacterial growth

Biofilms of MR10 were formed in BHIN for 24 h at 37 °C in the presence or absence of proteinase K, DNase I, dispersin B, or RNase A. The concentrations of these enzymes were the same as those used in the biofilm-quantification assay described below. Bacterial cells in the biofilm cultures (including biofilm and planktonic cells) were harvested, diluted with PBS, and spread on TSB agar. After incubation at 37 °C overnight, the number of colonies was counted, and the colony-forming units (CFUs) per 1 mL were calculated.

The growth curve of MR10 was measured in BHIN supplemented with or without RNase A (80 μg/mL) in a 96-well plate under static conditions for 24 h at 37 °C. The absorbance of each well at 600 nm was measured using an incubation reader (Scinics Corporation, Tokyo, Japan).

### Biofilm formation and quantification

Biofilm formation and quantification were performed as previously reported^31,44–46^. The bacterial strains were grown overnight at 37 °C in BHI medium. The cultures were diluted 1000-fold in BHIG, BHIN, BHINC, or RPMIG, and the suspensions were incubated at 37 °C for the indicated periods under static conditions. Biofilms in 96-well plates (Corning, Corning, NY) were washed twice with PBS (150 µL per well) and stained with 0.05% CV (150 µL per well) for 3 min at 25 °C. The wells were washed once with 150 µL PBS, and the absorbance at 595 nm was measured using an Infinite F200 Pro Microplate Reader (Tecan, Männedorf, Switzerland) to quantify the biofilm. To investigate the inhibition of biofilm formation by degradative enzymes, enzymes were added directly to the biofilm cultures in 96-well plates at the onset of biofilm formation. To examine biofilm dispersal, enzymes were added to 24-h biofilms formed at 37 °C in 96-well plates and incubated for 30 min at 37 °C before washing. The final enzyme concentrations were 16 µg/mL for dispersin B, 100 U/mL for DNase I, 100 µg/mL for proteinase K, and 80 µg/mL for RNase A in biofilm cultures. If required, lower concentrations of RNase A (5 and 10 µg/mL) and dispersin B (0.08 and 0.16 µg/mL) and their combinations were used to investigate their synergistic effect. All the enzymes were used without buffer. As a control, the biofilms were left untreated (neither enzyme nor buffer was added).

RNasin (Promega, Madison, WI) was used to inhibit the ribonuclease activity of RNase A. MR10 biofilms were formed in BHIN in 96-well plates for 24 h at 37 °C. The biofilms were washed once with PBS (200 µL per well) and treated with RNase A (0.8 µg/mL in PBS) for 30 min at 37 °C or left untreated. If required, native or heat-denatured (for 5 min at 105 °C) RNasin (80 U/µL) was added. The residual surface-attached cells were washed twice with PBS (200 µL per well) and stained with 0.05% CV (200 µL per well) for 3 min at 25 °C. The wells were washed once with 200 µL PBS, and the absorbance at 595 nm was measured using a microtiter plate reader.

Cells were grown in RPIMG medium to examine the biofilm-promoting effect of synthesized oligonucleotides or purified nucleic acids, which were added at the indicated concentrations at the beginning of biofilm growth. The MR10 biofilms formed in RPMIG in 96-well plates peeled off easily compared to those formed in BHIN. Therefore, changes were made to the washing and staining procedures described above. The biofilms were washed once with PBS (100 µL per well), stained with 0.05% CV (100 µL per well) for 3 min at 25 °C, and then washed once with 100 µL PBS. The absorbance at 595 nm was measured using a microplate reader.

### Biofilm formation under flow conditions

A 10 cm × φ 4.0–2.0 mm polyurethane tube (As One, Osaka, Japan) was autoclaved and subsequently connected to a syringe pump (SPE-1, As one). The tube was filled with a 1000-fold diluted overnight culture of MR10 in PNG supplemented with or without purified human RNA (1.25 μg/mL). After incubation for 1 h at 37 °C, PNG supplemented with or without human RNA (1.25 μg/mL) was pumped into the tube at a rate of 5.88 μL/min for 24 h at 37 °C. The biofilms formed in the tube were stained with 0.05% CV for 3 min at 25 °C and subsequently washed three times with PBS.

### Biofilm-matrix extraction

The biofilm matrix was extracted as described previously^26^. Briefly, staphylococcal biofilms were formed in 10 mL BHIG or BHIN in a 15 mL conical tube (Corning) for the indicated periods (4–24 h). After removing the supernatants, the tubes were centrifuged again, and the residual solutions were removed. The pellets were resuspended in 100 µL of 1.5 M NaCl and centrifuged at 5000 × *g* for 10 min at 25 °C. The supernatants were transferred to 1.5 mL tubes and centrifuged to remove all the bacterial cells. The supernatants were then passed through a 0.2-µm pore filter. The filtrates were used as biofilm-matrix fractions.

### Electrophoresis

Nucleic acids in the isolated biofilm matrix were separated by 1.5% (w/v) agarose gel electrophoresis or urea-denatured polyacrylamide gel electrophoresis using SuperSep RNA (Wako). If required, the samples were treated with dispersin B (16 µg/mL), DNase I (100 U/mL), proteinase K (100 µg/mL), or RNase A (80 µg/mL) for 30 min at 37 °C before loading onto the gel. After electrophoresis, nucleic acids were visualized by staining with ethidium bromide according to the conventional procedure and detected using an LAS 4000 luminescent image analyzer (GE Healthcare, Buckinghamshire, UK). All gel images were processed in parallel and derived from the same experiment. Original gel images are shown in the Supplementary Information.

### RNA purification

The biofilm matrix extracted from the MR10 biofilm was treated with dispersin B (80 µg/mL) and DNase I (400 U/mL) to digest the polysaccharides and DNA, respectively. After 2 h at 37 °C, RNA was purified from these samples using ISOGEN-LS (Wako) and used for RNA-seq analysis.

For the biofilm-promotion assay and RNA-seq analysis, RNA in 250 µL BHI medium was purified using 750 µL of ISOGEN-LS. Isopropanol-precipitated RNA was dissolved in 50 µL of double-distilled water (DDW). The partially purified RNA was further purified using 150 µL of ISOGEN-LS. After isopropanol precipitation, the RNA was dissolved in 10 µL of DDW. The purified RNA (BHI–RNA) was used for the biofilm-promotion assay.

Total bacterial RNA was extracted from the MR10. The MR10 cells were grown overnight at 37 °C in the BHI medium. The culture was diluted 100-fold in 100 mL RPMI, in which no nucleic acids were present. The suspension was incubated overnight at 37 °C and centrifuged at 5000 × *g* for 10 min at 25 °C. The pellets were resuspended in 950 µL of supernatant. The bacterial suspension was treated with 50 µL of lysostaphin (100 µg/mL, Wako) for 30 min at 37 °C. RNA was then extracted from the cell lysate using 750 μL of ISOGEN-LS according to the manufacturer’s protocol. Finally, the extracted RNA was dissolved in 100 μL of DDW for use in the biofilm-formation assay.

Human crude RNA (approximately 1000 ng/μL) was extracted from 250 μL of total human blood using 750 μL of ISOGEN-LS according to the manufacturer’s protocol. Then, 25 μL of 10× DNase I buffer composed of 400 mM Tris-HCl (pH 7.9), 100 mM NaCl, 60 mM MgCl_2_, 10 mM CaCl_2_ (Roche), and 12.5 μL of DNase I (Roche) were added to 215.5 μL of the extracted RNA. The mixture was incubated at 37 °C for 1 h. Then, RNA from 250 μL of the solution was purified again using 750 µL of ISOGEN-LS. Finally, the purified RNA was dissolved in 100 μL of DDW for use in the biofilm-formation assay. Blood was drawn from the veins of healthy volunteers using a conventional method after obtaining informed consent. The blood samples were stored at –80 °C until use. The collection and use of human-derived specimens were approved by the ethics committee of the Jikei University School of Medicine [Approval No. 21–104 (582)].

### RNA-seq

A library of RNA purified from the biofilm matrix of MR10 was prepared using the TruSeq Small RNA Sample Prep Kit (Illumina, San Diego, CA) and sequenced using Illumina HiSeq with paired-end reads of 100 nucleotides (Hokkaido System Science, Hokkaido, Japan). The sequenced fragments were trimmed using Cutadapt, a program that removes adapter sequences from DNA-sequenced reads. The trimmed sequences were then merged using fastqjoin, a program that merges overlapping paired-end reads. The merged sequences were mapped to the *S. aureus* Mu50 genome^33^ with Geneious 11.1.5 (Biomatters, Auckland, New Zealand) and CLC Genomics workbench (Filgen, Bristol, UK). As required, we counted the sequences of RNA-seq using the script we created (Supplementary Note).

For comparison of RNA sequences between BHI medium and the biofilm-matrix fraction of MR10, the sequence library was prepared according to the TruSeq Small RNA Preparation Kit protocol (Illumina) and sequenced using Illumina HiSeq 3000 with paired-end reads. The RNA sequences were analyzed as described above.

### RT-PCR

We performed RT-PCR using RNA purified from BHI medium and the isolated biofilm matrix of MR10 as a template. Specific primers were designed based on the RNA-seq data (Supplementary Table 1). cDNA was obtained by reverse transcription using the PrimeScript kit (Takara Bio, Otsu, Japan). The resultant cDNA was amplified by PCR using the KOD polymerase (Toyobo, Osaka, Japan). The PCR products were verified using agarose gel electrophoresis. If necessary, we cloned the amplified fragment into the pGEM-T easy vector (Promega) and sequenced it by Eurofins Genomics (Tokyo, Japan).

### Ethanol precipitation

Nucleic acids from autoclaved BHI medium were obtained through ethanol precipitation. Briefly, 1 mL of 100% ethanol (Wako) was added to 400 µL of BHI medium, and the mixture was incubated for 10 min at 25 °C and then centrifuged at 20,000 × *g* for 20 min at 4 °C. The supernatant was removed, and the pellets were washed once with 1 mL of 70% ethanol and resuspended in 50 µL of DDW.

### RNA pull-down assay

MR10 wild-type and ∆*icaA* biofilms were grown for 24 h at 37 °C in 1 mL of BHIN in a 1.5 mL tube. If required, dispersin B (16 µg/mL) or proteinase K (100 µg/mL) was added at the beginning of biofilm formation. The tube was centrifuged at 8000 × *g* for 10 min at 25 °C, the supernatant was removed, and the pellets were used as the biofilm. The MR10 cells were grown in BHI medium for 24 h at 37 °C. The culture supernatant was filtered and the filtrate was used as the RNA solution. The biofilms and RNA solution were mixed, and a small aliquot of the mixture was used as the input fraction. The residual mixture was immediately centrifuged at 8000 × *g* for 10 min at 25 °C. The supernatant was collected as the nonbound RNA fraction. The pellets were further suspended in 1.5 M NaCl to dissociate RNA from the biofilms. The resulting suspension was used as the bound RNA fraction. These fractions were analyzed using agarose gel electrophoresis to detect RNA. This procedure is illustrated in Supplementary Fig. 4.

### Extraction and purification of polysaccharides from *S. aureus*

Polysaccharides were purified from *S. aureus* using the method described by Maira-Litrán et al.^47^ with modifications. Briefly, a single colony of MR10 on a BHI agar plate was inoculated in 240 mL of BHIN broth and incubated for 24 h at 37 °C. Polysaccharide-associated bacterial cells were collected via centrifugation at 8000 × *g* at 25 °C. Five-hundred microliters of 1.5 M NaCl were added to the pellets and suspended by sonication. To remove the bacterial cells, the suspension was centrifuged at 5000 × *g* for 10 min at 25 °C, and the supernatant was collected. This step was repeated, and the supernatant was further centrifuged at 20,000 × *g* for 5 min at 25 °C. The resulting supernatant containing polysaccharides (430 µL) was transferred to a new 1.5 mL tube and supplemented with 50 µL of 10× DNase I (Roche), 10 µL of DNase I (200 U/mL), and 10 µL of RNase A (200 µg/mL). This mixture was incubated for 1 h at 37 °C and further treated with proteinase K (200 µg/mL) overnight at 37 °C. To denature proteinase K, this crude polysaccharide solution was incubated at 95 °C for 10 min and filtered through a 0.2-µm pore filter. The filtrate was subjected to size-exclusion chromatography with Superdex 200 10/300 GL column (GE Healthcare) equilibrated with running buffer containing 50 mM Tris-HCl (pH 7.4) and 1.5 M NaCl. Polysaccharides eluted in the void volume monitored by absorption at 206 nm were detected by SDS-PAGE as the band at the top of polyacrylamide gels^26^ and via lectin-blotting. The fractions containing the polysaccharides were pooled and stored at 4 °C until further use.

### Complementation assay

A plasmid for the complementation of *icaA* deletion was constructed using *E. coli*–*S. aureus* shuttle vector pLC1, as previously described^46,48^ (Table 1). Briefly, pLC1 was linearized by inverse PCR using KOD Plus (Toyobo) and the primers pLC1-F and pLC1-R (Supplementary Table 1). The *icaADBC* operon with the 30-bp upstream region containing the ribosome-binding site of *icaA* was amplified by PCR from genomic DNA using the primers comp_ica_F and comp_ica_R (Supplementary Table 1). The amplified fragment was cloned into linearized pLC1 using the GeneArt Seamless Cloning and Assembly Kit (Thermo Fisher Scientific) according to the manufacturer’s protocol. The constructed plasmids pLC1::*ica* and pLC1 were used to transform *S. aureus* RN4220^28^ through electroporation. After purification, the plasmids were introduced into MR10 Δ*icaA* cells through electroporation. Complementary strains harboring pLC1 or its derivatives were cultured on BHI agar containing 10 µg/mL Cm. A single colony on the plate was inoculated into BHI broth containing 10 µg/mL Cm and grown at 37 °C overnight. The culture was diluted 1000-fold into BHIN and incubated at 37 °C for 24 h in 96-well plates or plastic dishes to form biofilms. During biofilm formation, no antibiotics were added to the medium, to avoid the negative effects of antibiotics on biofilm formation.

### CLSM

Overnight cultures of MR10 wild type and ∆*icaA* were diluted 1000-fold in BHINC and 100 µL of the suspension was incubated with 19R-Alexa 488 (25 µg/mL) at 37 °C for 24 h under static conditions on a 35 mm polystyrene dish (Thermo Fisher Scientific). After incubation, the supernatant was removed by pipetting. Fifty microliters of 10% paraformaldehyde were mounted on the biofilm to fix the biofilm for 10 min at 25 °C. Then, 50 mM NH_4_Cl was added to the sample to quench the paraformaldehyde and immediately removed. The samples were washed with 50 µL of PBS. For polysaccharide staining, the fixed biofilm was incubated with WGA-Alexa 594 (5 µg/mL) for 20 min at 37 °C. After incubation, excess WGA-Alexa 594 was removed, and the biofilm was washed with 50 µL of PBS. DNA was then stained with 50 µL of DAPI solution (1 µg/mL) for 5 min at 25 °C. A coverslip was placed on the sample. The biofilm and extracellular structures were observed with LSM880 in the Airyscan super-resolution mode (Carl Zeiss, Oberkochen, Germany). All the images were digitalized at a resolution of 16 bits into an array of 512 × 512 pixels with four averages. The optimal conditions of fluorescent samples were obtained using an argon laser (wavelength 488 nm, 3%), diode 405–30 laser (405 nm, 1%), and DPSS 561–10 laser (561 nm, 1%) with a Plan-Apochromat 63×/1.4 Oil DIC M27 (Carl Zeiss).

### Surface plasmon resonance analysis of purified polysaccharides and RNA

We analyzed the direct interaction between polysaccharides and RNA using a BIAcore T200 surface plasmon resonance analyzer (GE Healthcare). To prepare ligands, biotinylated 19 R was diluted with HBS-EP buffer, composed of 10 mM HEPES (pH 7.4), 150 mM NaCl, 3 mM EDTA, and 0.05% (v/v) surfactant P, and immobilized on a streptavidin-coated Series S Sensor Chip SA (GE Healthcare) at a flow rate of 10 µL/min for 60 s. All the datasets were collected at 25 °C.

Two-hundred microliters of purified polysaccharides were dialyzed using Slide-A-Lyzer 10 K MWCO (Thermo Fisher Scientific) in 500 mL of HBS-EP overnight at 4 °C. The dialyzed polysaccharides were diluted in HBS-EP buffer and subjected to analytes at a flow rate of 30 µL/min for 30 s in manual mode. Because the molecular-size distribution of polysaccharides purified from the biofilm matrix was vast, the molar concentration of the polysaccharides was difficult to determine. Dispersin B was diluted in HBS-EP to a final concentration of 16 µg/mL and used to digest the bound polysaccharides at a flow rate of 30 µL/min for 30 s.

### Statistical analysis

The experiments were performed at least three times. The data were analyzed with the EZR application^49^. Statistical significance was set at *P* < 0.05. Statistical corrections were made using the Bonferroni method.

### Reporting summary

Further information on research design is available in the Nature Research Reporting Summary linked to this article.

## Supplementary information

Supplementary Information

Reporting Summary

## Data Availability

The data associated with the paper are available in the DDBJ Sequence Read Archive (DRA) (https://www.ddbj.nig.ac.jp/dra/index-e.html) under the accession numbers DRA006074 and DRA007403.

## Code availability

Script used to count RNA sequences in the NGS data is provided as Supplementary Note.
