# Supplementary material for: Staphylococcus aureus utilizes environmental RNA as a building material in specific polysaccharide-dependent biofilms
Source: NPJ Biofilms Microbiomes. 2022 Apr 4;8:17. doi: 10.1038/s41522-022-00278-z (PMC8980062; doi:10.1038/s41522-022-00278-z)
Supplement: Supplementary file 1 — Supplementary Information [file 41522_2022_278_MOESM1_ESM.pdf]

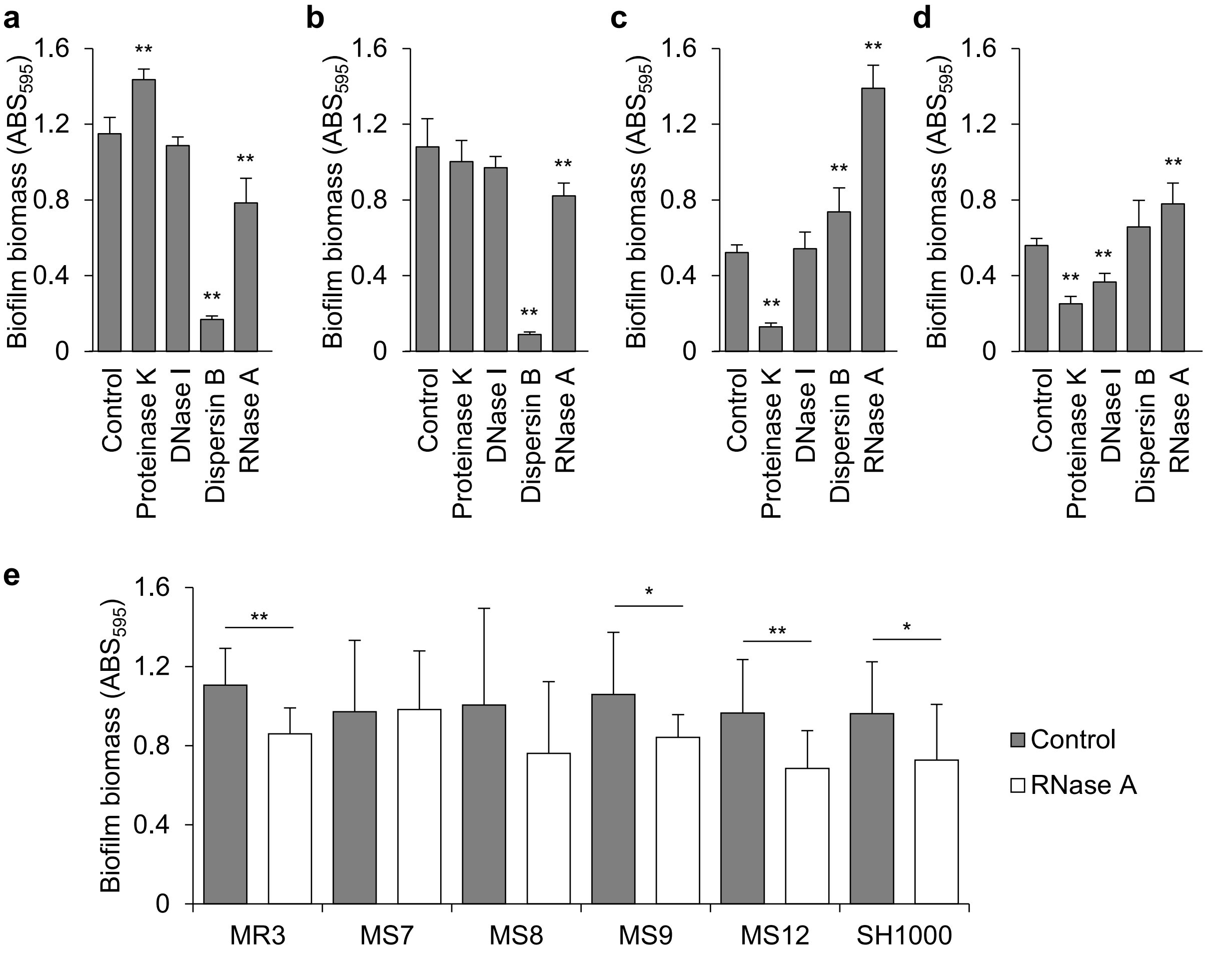

Supplementary Figure 1. Effects of enzymes on polysaccharide-dependent and -independent staphylococcal biofilms. (**a, b**) The *S. aureus* laboratory strain RN4220 was cultured in BHIN. (**c, d**) The clinically isolated *S. aureus* strain USA300 was cultured in BHIG. (**e**) The indicated polysaccharide-producing strains were cultured in BHIN. (**a, c**) Inhibition of biofilm formation by the indicated enzymes was examined. These enzymes were added to the media at the onset of biofilm formation. (**b, d, e**) Biofilm dispersal was analyzed. The 24-h biofilms were treated with the indicated enzymes. As controls, the biofilms were left untreated. The data are presented as the mean and standard deviation (error bar) of three independent experiments. $^{**}P < 0.01$.

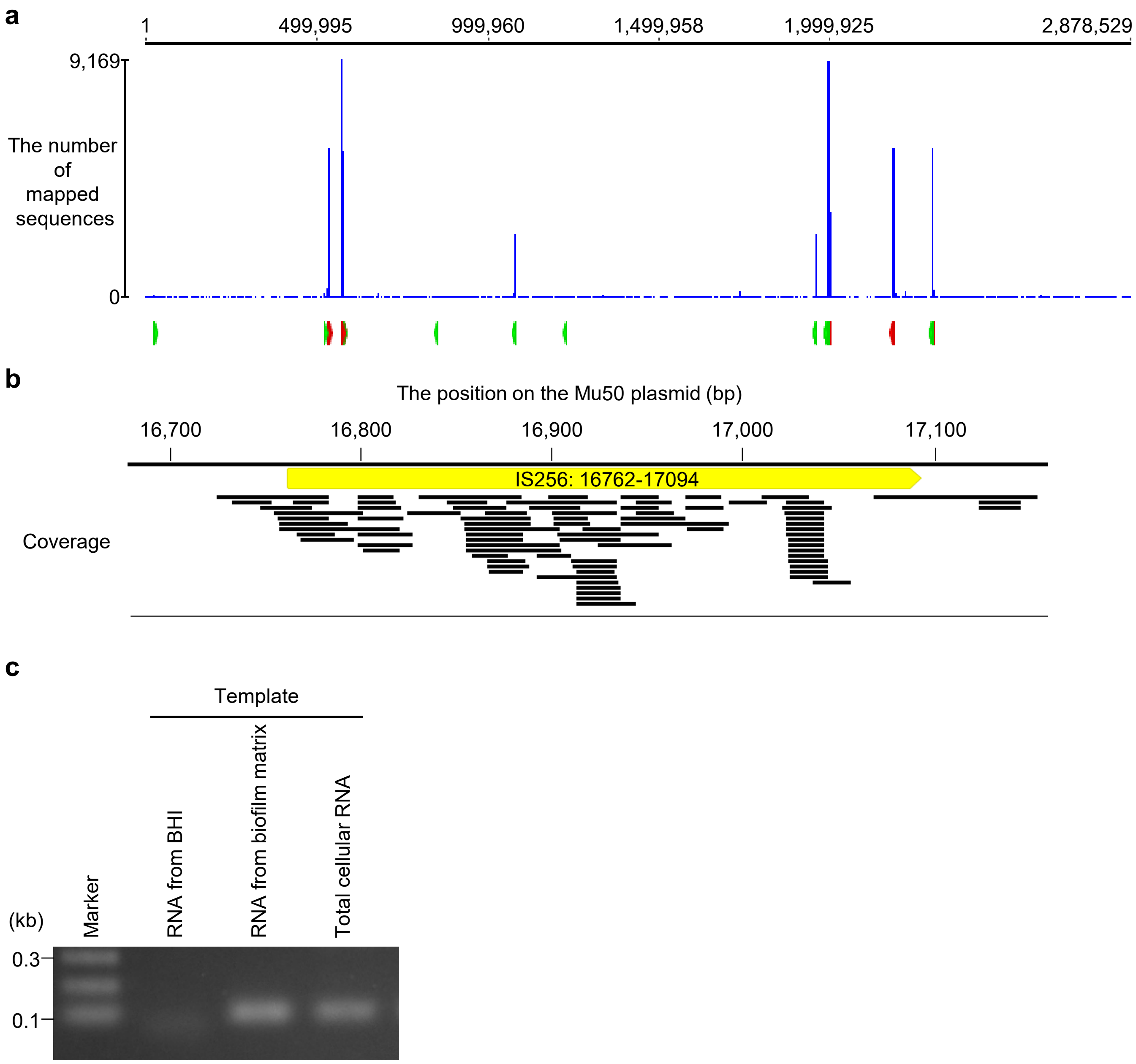

Supplementary Figure 2. **Extracellular RNAs derived from *S. aureus*.** (**a**) The 110,111 sequences obtained through RNA-seq were mapped to the *S. aureus* Mu50 genome. The green and red markers represent the positions and directions of tRNA and rRNA, respectively. (**b**) RNA-seq data showing the coverage of cDNA reads over the IS256 gene in the plasmid of *S. aureus* Mu50 (position 16,762–17,094). (**c**) RT-PCR detection of the IS256 RNA fragment that can be released from *S. aureus* MR10 into the extracellular milieu. The primers were designed based on the IS256 sequence. The templates for RT-PCR were RNAs purified from BHI medium and MR10 24-h biofilm matrices, and total cellular RNAs extracted from MR10 biofilm cells grown in BHIN. Total RNA was extracted using the RNeasy Maxi kit (Qiagen, Hilden, Germany) according to the manufacturer’s protocol. Amplicons were analyzed using agarose gel electrophoresis.

a

Sequence (5' to 3')	BHI Rank	BHI Count	BHI (%)	Biofilm matrix Rank	Biofilm matrix Count	Biofilm matrix (%)
TCGTTAGTTCAATTCTGACAGGTGGCACCA	1	6,590,141	9.90	1	5,086,258	5.50
TCGTTAGTTCAATTCTGACAGGTGGCACC	2	3,644,796	5.48	2	2,551,844	2.76
TTAGGGGTTCGAGCCCCCTACAGGGCTC	3	2,146,022	3.22	4	1,543,944	1.67
TCAGGGGTTCGAGCCCCCTATGAGGAGCC	4	1,139,884	1.71	3	2,094,124	2.26
TCTCCGGTTCGATTCCGGACTCGTCCACC	5	888,413	1.34	12	334,087	0.36
CGTTAGTTCAATTCTGACAGGTGGCACCA	6	812,650	1.22	9	677,174	0.73
TTAGGGGTTCGAGCCCCCTACAGGGCT	7	807,488	1.21	8	679,166	0.73
TTAGGGGTTCGAGCCCCCTACAGGGCTCC	8	629,269	0.95	11	433,164	0.47
TCGTTAGTTCAATTCTGACAGGTGGCAC	9	628,579	0.94	16	315,793	0.34
TCCTGTGTTCGATCCACAGAATTCGCACC	10	553,212	0.83	49	116,597	0.13
TCGTTAGTTCATTTCTGACAGGTGGCACCA	11	531,990	0.80	14	322,236	0.35
CGTTAGTTCAATTCTGACAGGTGGCACC	12	417,785	0.63	13	326,441	0.35
CTCCGGTTCGATTCCGGACTCGTCCACC	13	385,361	0.58	39	139,829	0.15
TCCTGTGTTCGATCCACAGAATTCGCACCA	14	380,047	0.57	56	95,449	0.10
TTAGGGGTTCGAGCCCCCTACAGGGC	15	327,602	0.49	23	228,043	0.25
TCAGGGGTTCGAGCCCCCTATGAGGAGC	16	309,276	0.46	6	902,123	0.98
TCGTTAGTCAATTCTGACAGGTGGCACCA	17	298,486	0.45		NA	
TCCTTAGTTCGATCCTGAGTGCGAGCTC	18	285,184	0.43	20	249,433	0.27
TCGCTAGTTCAATTCTGGCAGGTGGCACCA	19	268,027	0.40	38	140,829	0.15
TCGTTAGTTCATTTCTGACAGGTGGCACC	20	262,307	0.39	36	146,790	0.16

b

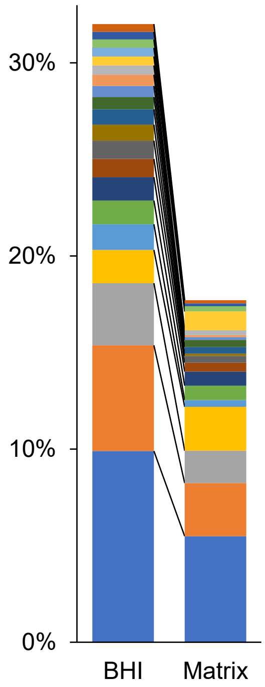

Supplementary Figure 3. Comparison of RNA contents between BHI and biofilm matrix. (a) Sequences of RNAs purified from BHI medium and the biofilm matrix of MR10 were analyzed by RNA-seq. The top 20 abundant RNAs in the medium are shown. Abundant RNA sequences found in the biofilm matrix (termed 19R) is colored in magenta. (b) A graphical representation of data in a.

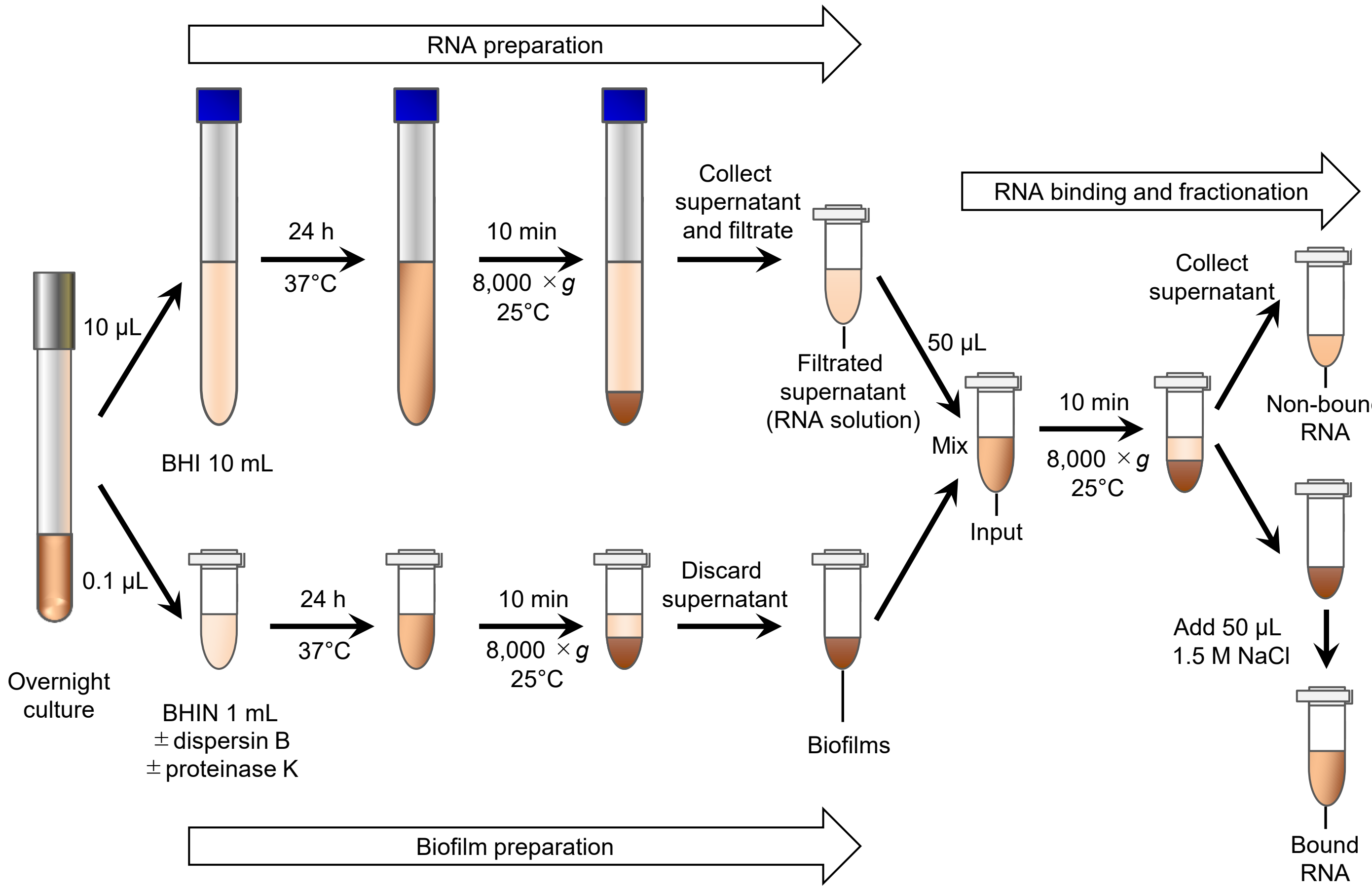

Supplementary Figure 4. Scheme of RNA pull-down. RNA was prepared from a 24-h culture of MR10 in BHI medium (upper process) and the culture supernatant was filtered and used as an RNA solution. In parallel, MR10 biofilms were formed in BHIN for 24 h at 37°C and harvested in a tube (lower process). If required, the biofilms were treated with dispersin B or proteinase K for 30 min at 37°C. The pelleted fraction was used as the biofilm cell. The resultant RNA solution and biofilm cells were mixed and immediately centrifuged, and the supernatant was used as the non-bound RNA fraction. The residual pellet, upon being resuspended in 1.5 M NaCl, was used as a bound RNA fraction.

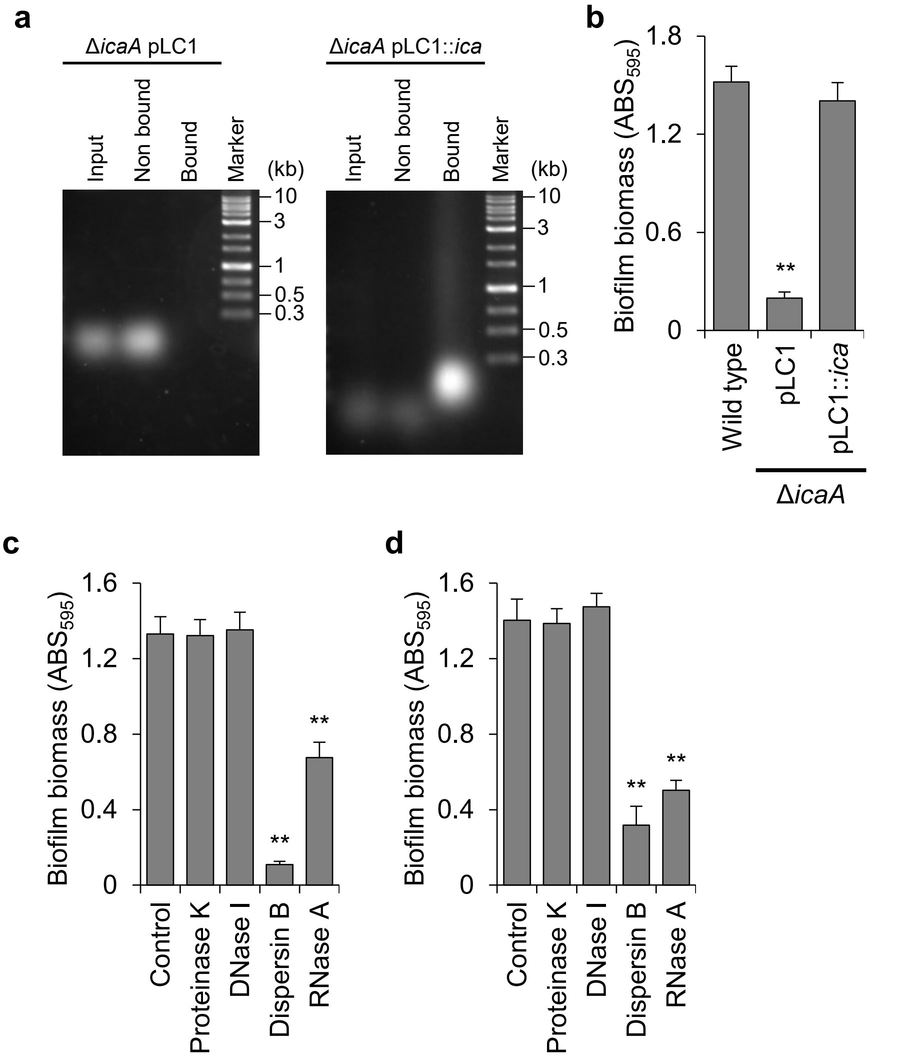

Supplementary Figure 5. Complementation of the deletion of Δ*icaA* in the strain MR10. (**a**) RNA pull-down was performed as illustrated in Supplementary Figure S3. Biofilm-associated and non-associated RNA from MR10 Δ*icaA* pLC1 (empty plasmid) and MR10 Δ*icaA* pLC1::*ica* were detected using agarose gel electrophoresis. (**b**) Biofilms of MR10 Δ*icaA* pLC1 and MR10 Δ*icaA* pLC1::*ica* were formed in the BHIN for 24 h. The biofilm biomasses of MR10 wild type are also shown as control (see Figure 1). (**c**) MR10 Δ*icaA* pLC1::*ica* 24-h biofilms were treated with the indicated enzymes for 30 min or left untreated (Control). (**d**) MR10 Δ*icaA* pLC1::*ica* biofilms were formed in the absence (Control) or presence of the indicated enzymes for 24 h. Biofilm biomasses are presented as the mean and standard deviation (error bar) of three independent experiments. $^{**}P < 0.01$.

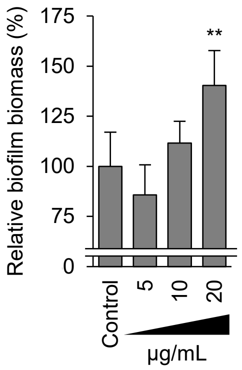

Supplementary Figure 6. Promotion of biofilm formation by synthesized RNA. Synthesized RNA named 19R (Supplementary Table S1) was added to RPMIG at the indicated concentrations from the onset of biofilm formation by the strain MR10. The biofilm biomass was measured after incubation for 24 h. The data are presented as the mean and standard deviation (error bar) of the relative biofilm biomass from three independent experiments. The biofilm biomasses in the absence of 19R (Control) were defined as 100%. **P < 0.01.

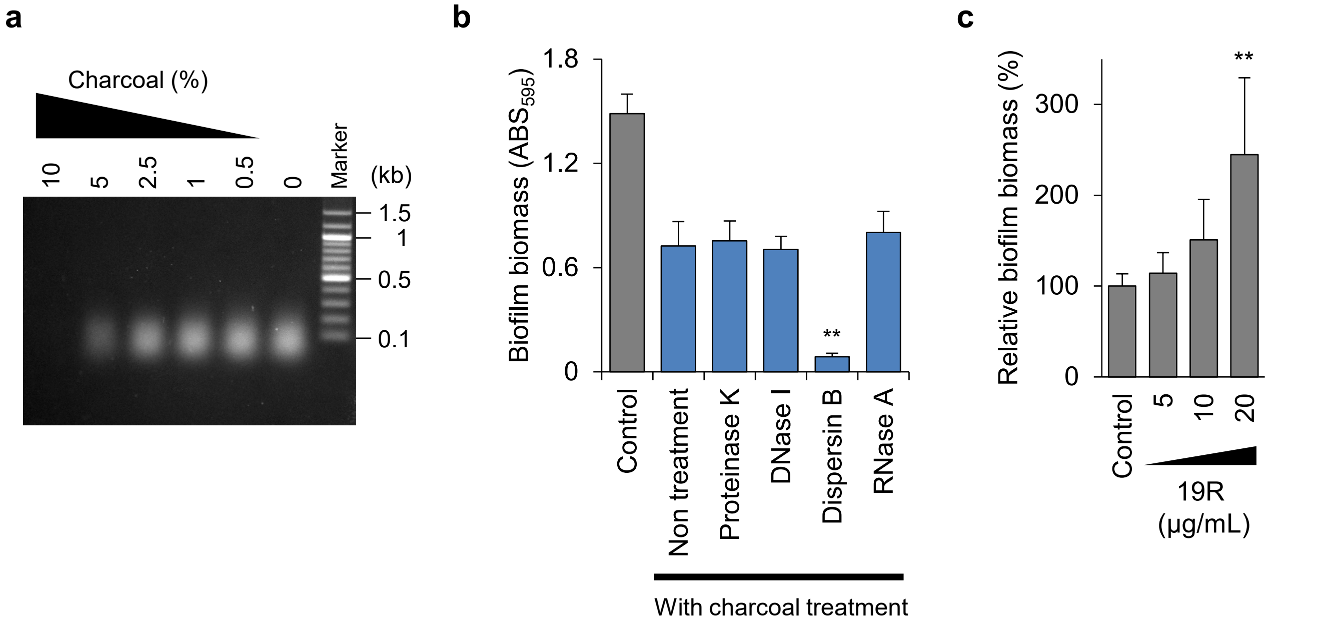

Supplementary Figure 7. Biofilm formation in the RNA-depleted BHI medium. (**a**) The indicated concentrations of activated charcoal and BHIN were mixed and filtrated. Then, the nucleic acid contents in the media were analyzed using agarose gel electrophoresis. (**b**) Biofilms of the strain MR10 were formed in the BHIN treated with 10% activated charcoal (BHINC). The 24-h biofilms were treated with the indicated enzymes for 30 min at 37°C and quantified. The data are presented as the mean and standard deviation of three independent experiments. The biomasses of the biofilms formed in BHIN without treatment with charcoal are also shown as control (see Figure 1). (**c**) The synthesized RNA named 19R was added into BHINC at the indicated concentrations from the onset of biofilm formation. The biofilm biomass was measured after incubation for 24 h. The data are presented as the mean and standard deviation (error bar) of relative biofilm biomass from three independent experiments. The biofilm biomasses in the absence of 19R (Control) were defined as 100%. ***P* < 0.01.

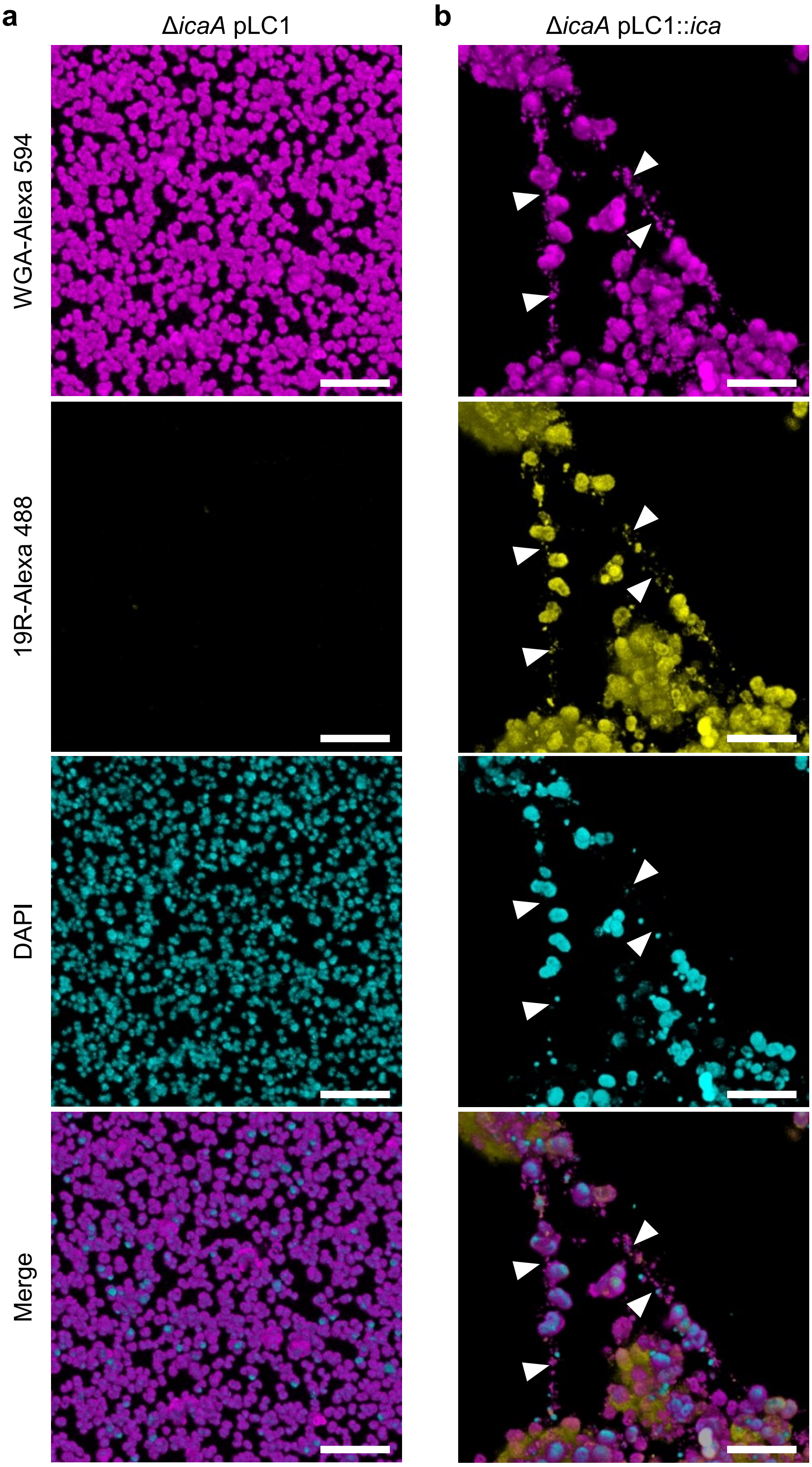

Supplementary Figure 8. Localization of polysaccharides and RNA in the biofilms of MR10 Δ*icaA* harboring pLC1 and pLC1::*ica*. MR10 Δ*icaA* pLC1 (**a**) and pLC1::*ica* (**b**) were cultured in BHINC supplemented with 19R-Alexa 488 for 24 h at 37°C. The biofilms were stained with WGA-Alexa 594 and DAPI. Subsequently, the biofilms were observed using CLSM (with Airyscan unit). WGA-Alexa 594 and DAPI stained the polysaccharides and the cell wall (magenta) and DNA (cyan), respectively. 19R-Alexa 488 indicates localization of RNA (yellow). The merged images are also shown. The scale bars correspond to 5 μm. The arrowheads indicate co-localization of the polysaccharides and RNA.

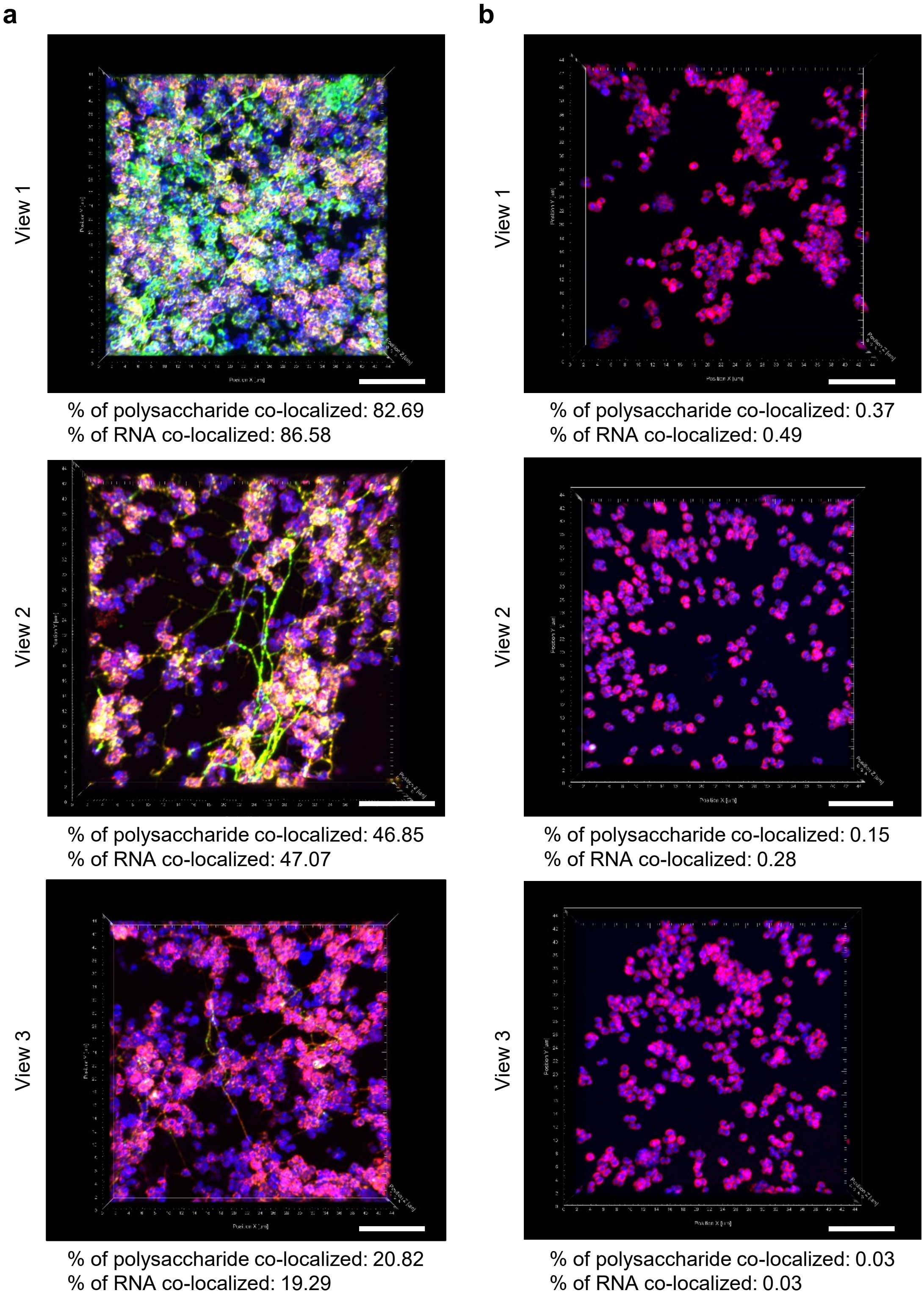

Supplementary Figure 9. Image analysis for co-localization of RNA and polysaccharides. Biofilms of the MR10 wild-type (**a**) and Δ*icaA* (**b**) strains were observed using high-resolution CLSM, as shown in Figure 5c. Representative three views of each strain are shown. View 2 consists of low magnification images of Figure 5c. WGA-Alexa 594 and DAPI stained the polysaccharides and the cell wall (magenta) and DNA (blue), respectively. 19R-Alexa 488 indicates localization of RNA (green). Co-localization of the RNA and polysaccharides was analyzed with Imaris (Oxford Instruments, Abingdon, UK). The bars correspond to 10 μm.

Supplementary Table 1. Oligonucleotides used in this study.

Name	Sequence (from 5' to 3')	Description
**Primer**		
f9959-F	GAAAGCACCGTTTCCCGTC	A specific forward primer to amplify the fragment identified by RNA-seq
f9959-R	AGATTGCAGCACCTGAGTTTC	A specific reverse primer to amplify the fragment identified by RNA-seq
f8400-F	TCATTTGTATACGACTTAGATGTACAACGG	A specific forward primer to amplify the fragment identified by RNA-seq
f8400-R	ACAAATCAGACAACAAAGGCTTAATCTC	A specific reverse primer to amplify the fragment identified by RNA-seq
Rf235287-F	TAATTGAACGACTGAATCAAGAAGTACG	A specific forward primer to amplify the IS256 fragment
Rf235287-R	GAACGGCTCCAATTAAGCGA	A specific reverse primer to amplify the IS256 fragment
gem2928-2949	CAAGGCGATTAAGTTGGGTAAC	Designed from pGEM-T vector for sequencing of cloned DNA fragments
gem231-209	GTATGTTGTGTGGAATTGTGAGC	Designed from pGEM-T vector for sequencing of cloned DNA fragments
icaAKO_F	GGGGACAAGTTTGTACAAAAAAGCAGGCTGAATTGATATAAAGCATCAATTGAATAG	A specific forward primer to amplify the upstream region of *icaA* in MR10
icaAKO_R	GGGGACCACTTTGTACAAGAAAGCTGGGTCAGAAAATTCGCTTTTCTTACAC	A specific reverse primer to amplify the downstream region of *icaA* in MR10
SOE_icaA_F	ATTGAACAAGAAGCCTGACATAAATAATCATCGCTAAATATTGTAAGAGA	A specific forward primer to amplify the downstream region of *icaA* in MR10
SOE_icaA_R	TCTCTTACAATATTTAGCGATGATTATTTATGTCAGGCTTCTTGTTCAAT	A specific reverse primer to amplify the upstream region of *icaA* in MR10
pLC1-F	CTGCAGCCAAGCTAGCTTGGC	A specific forward primer to amplify and linearize pLC1
pLC1-R	CCCGGGAGATCTGATATCAAGCTTATTTTA	A specific forward primer to amplify and linearize pLC1
comp_ica_F	TTGATATCAGATCTCCCGGGAACCTAACTAACGAAAGGTAGGTAAAGAAA	A specific forward primer to amplify the *ica* operon containing the ribosome-binding site of *icaA*
comp_ica_R	CCAAGCTAGCTTGGCTGCAGCCACCGCGTGTTTTTAACAT	A specific forward primer to amplify the ica operon containing the ribosome-binding site of *icaA*
19R	CGUUAGUUCAAUUCUGACA	The most highly conserved sequence in external RNAs identified by RNA-seq

Supplementary Note. Counting script. This short script named "Genomcount" was written in C# to count all sequences in the NGS data. The trimmed RNA-seq data in fastq format were used as inputs for the script. The script outputs sequences and the number of counts in descending order of number.

```
using System.IO;
using System.Collections.Generic;
using System.Linq;

public class GenomCount
{

	public class GenomInfo
	{
		public GenomInfo(string genom, int count = 0) { this.genom = genom; this.count = count; }
		public string genom;
		public int count;
	}


	public static void Main(string[] args)
	{
		TextReader input =
			(args.Length == 0) ? Console.In :
				new StreamReader(args[0], System.Text.Encoding.GetEncoding("Shift_JIS"));

		ReadGenoms(input)
			.GroupBy(it => it)
			.Select(it => new GenomInfo(it.First(), it.Count()))
			.OrderByDescending(it => it.count)
			.Select(it => it.count.ToString() + "," + it.genom)
			.ToList()
			.ForEach(it => Console.WriteLine(it));
	}


	static IEnumerable<string> ReadGenoms(TextReader tr)
	{
		do
		{
			var line = tr.ReadLine();
			while (!line.StartsWith("@"))
			{
				if (line == null)
					yield break;
				line = tr.ReadLine();
			}

			var genom = tr.ReadLine();
			tr.ReadLine(); //skip
			tr.ReadLine(); //skip
			yield return genom;
		} while (tr.Peek() != -1);
	}

}
```
